# Supplementary material for: Intestinal Helminths in Wild Rodents from Native Forest and Exotic Pine Plantations (Pinus radiata) in Central Chile
Source: Animals (Basel). 2021 Feb 3;11(2):384. doi: 10.3390/ani11020384 (PMC7913297; doi:10.3390/ani11020384)
Supplement: Supplementary file 1 [file animals-11-00384-s001.pdf]

Table 1. Comparison of models used to test the effect of independent variables (habitat type, season and rodent hosts) on individual positivity for helminths in rodents according to their AICc values. Models also include degrees of freedom (df), AICc differences (ΔAICc) and Akaike weights (w_r).

Models	df	AICc	ΔAICc	w_r
Season + species	6	930.0	0.00	0.729
Species	3	933.5	3.48	0.128
Habitat + season + species	8	933.7	3.65	0.118
Habitat + species	5	937.2	7.18	0.020
Season	4	940.5	10.46	0.004
Habitat + season	6	943.0	13.04	0.001

Table S2. Comparison of models used to test the effect of independent variables (habitat type, season and rodent species) on individual positivity for *Hymenolepis* sp. in rodents according to their AICc values. Models also include degrees of freedom (df), AICc differences (ΔAICc) and Akaike weights (w_r).

Models	df	AICc	ΔAICc	w_r
Species	3	482.1	0.00	0.731
Species + habitat	5	485.2	3.19	0.149
Species + season	6	487.3	5.29	0.052
Habitat	3	488.6	6.56	0.028
Habitat + season + species	8	490.3	8.25	0.012

Table S3. Comparison of models used to test the effect of independent variables (habitat type, season and rodent species) on individual positivity for *Physaloptera* sp. in rodents according to their AICc values. Models also include degrees of freedom (df), AICc differences (ΔAICc) and Akaike weights (w_r).

Models	df	AICc	ΔAICc	w_r
Season + species	6	301.9	0.00	0.577
Season + habitat	6	304.3	2.38	0.175
Habitat + season + species	8	304.6	2.71	0.149
Species	3	306.2	4.27	0.068
Habitat + species	5	308.7	6.86	0.019
Season	4	309.6	7.74	0.012
